# Carer's Attachment Anxiety, Stressful Life-Events and the Risk of Childhood-Onset Type 1 Diabetes

**DOI:** 10.3389/fpsyt.2021.657982

**Published:** 2021-05-25

**Authors:** Anja Turin, Klemen Dovč, Simona Klemenčič, Nataša Bratina, Tadej Battelino, Jasna Klara Lipovšek, Katarina Uršič, Yael Shmueli-Goetz, Maja Drobnič-Radobuljac

**Affiliations:** ^1^Centre for Mental Health, University Psychiatric Hospital Ljubljana, Ljubljana, Slovenia; ^2^Faculty of Medicine, University of Ljubljana, Ljubljana, Slovenia; ^3^Department for Endocrinology, Diabetes and Metabolic Disease, University Children's Hospital, University Medical Centre Ljubljana, Ljubljana, Slovenia; ^4^Anna Freud National Centre for Children and Families and Psychoanalysis Unit, University College London, London, United Kingdom

**Keywords:** attachment—a strong affectional bond, child/adolescent, stress, stressful life events, type 1 diabetes mellitus, etiology

## Abstract

**Background/Objective:** Type 1 diabetes (T1D) is among the most common chronic diseases in children/adolescents, and the incidence continues to rise worldwide. Different environmental factors have been evaluated in the etiology. In the present study, we investigated the role of attachment examining whether insecure attachment to carers or carers' own attachment insecurity was related to a higher risk of T1D in children.

**Methods:** We included 101 children with T1D (mean age 11.8 years), 106 healthy controls (11.6 years), and one of their carers. We assessed children's attachment using the Child Attachment Interview and carers' attachment using the Relationship Structures Questionnaire. We constructed binary multinomial logistic regression models using attachment to mothers, carers' attachment representations, and stressful life-events as T1D predictors.

**Results:** Higher carer attachment anxiety was associated with the child's T1D diagnosis (*p* < 0.05; *R*^2^ = 0.0613) while security of attachment to mothers showed no significant association. When mothers' education was included in the model, both attachment anxiety in higher educated mothers and stressful life events showed a significant association with the child's T1D (*p* < 0.001; *R*^2^ = 0.293).

**Conclusions:** Our findings suggest that higher attachment-related anxiety in carers with high education and stressful life events are associated with T1D in children.

## Introduction

Type 1 diabetes (T1D) is among the most common chronic diseases in children and adolescents. The incidence continues to rise worldwide by ~2–5% annually (1). T1D is a consequence of autoimmune destruction of pancreatic beta cells, which leads to a complete lack of insulin production (2). The environmental factors leading to the development of T1D in genetically susceptible individuals, either by triggering islet autoimmunity or progression from autoimmunity to overt T1D, have only been partially identified (3). Psychological stress is shown to be a possible risk factor by increasing insulin resistance leading to increased demand on beta cells and by directly influencing immune response and causing alterations at the hypothalamic-pituitary-adrenal axis (4). Major life events (e.g., the death of a close relative or divorce) and psychological strain in the family have been consistently identified as risk factors for islet autoimmunity and T1D in a number of studies, including large population-based prospective studies (5, 6). One psychological factor that may be implicated in the development and maintenance of T1D in childhood is the quality of attachment relationships. Based on the caregiver's availability and sensitive responsiveness in relation to their infant's bids for proximity-seeking at times of distress, internal models of relationships are formed that guide behavior and expectations in a largely unconscious way (7). The parent-child attachment relationship serves as a context in which an infant's emotions and stress are regulated: as an interpersonal biobehavioral stress regulatory system. Individual differences in infant attachment are thought to reflect distinctive strategies for responding to and coping with interpersonal challenges (8, 9), exposing insecurely organized individuals to the deregulated autonomic nervous system and exaggerated hypothalamic-pituitary-adrenal activity, which, in turn, produces increased and prolonged exposure to stress hormones. Such stress responses may have considerable implications for the development of diverse health-risk conditions, such as insulin resistance and hyperlipidemia (10).

The evidence for these possible associations to date, however, is scant. Ciechanowski and colleagues demonstrated that dismissing attachment was related to poor glycemic control in adults with T1D (11). Sepa et al. investigated the association of mothers' attachment insecurity with the induction or progression of diabetes-related autoimmunity in early childhood using interviews with 18 mothers of antibody-positive infants and 32 mothers of antibody-negative infants. Their results showed a larger proportion of insecure mothers in the antibody-positive group, although the association was not statistically significant. They concluded that if an association between mothers' attachment and diabetes-related autoimmunity in children exists, it was not very strong, acknowledging their small sample size as well as a generally imperfect correlation between mother and child attachment (12). To our knowledge, no studies have examined the role of the child's and the carer's attachment security in the development of T1D.

To address this, the present study was undertaken in order to test associations between insecure attachment of children to their carers, carers' attachment insecurity, and T1D in children. We assumed insecure attachment of children or their parents together with stressful life events would be related to children's T1D.

## Materials and Methods

### Participants and Procedures

All eligible children with T1D in Slovenia included in the Slovenian national childhood-onset T1D register and their parents/caregivers were invited to participate in this case-control observational study. An invitation to participate was sent by mail, followed by a personal phone call invitation from their diabetologist before attending one of the regular 3-monthly control visits.

The control group consisted of healthy pupils from 3rd to 9th grade from five randomly chosen primary schools from the entire country. The study was presented by one of the researchers or the school counselor during classes at school, and to the parents at the regular bi-yearly school meetings for parents. Parents were also given a written information sheet explaining the study. The invitation was followed by another inquiry to each invited class by the school counselors.

The main inclusion criteria were age between 8 and 15 years and diagnosis of T1D for over 1 year (the cases). The main exclusion criteria were intellectual disability and/or active psychosis and, in the controls, T1D.

The enrolment began in July 2015 and ended in December 2019. Participation was voluntary and anonymous, and all participants and/or their parents signed informed assents/consents prior to the enrolment. The interview recording and the assessments were completed at the University Children's Hospital (the cases) or at the school before the beginning of classes (control group). The protocol of the study was approved by the National Medical Ethics Committee of the Republic of Slovenia (No. 60/08/13). Trial Registration: ClinicalTrials.gov (NCT02575001).

### Measures

General demographic and family characteristics were acquired by a special questionnaire administered to the carers (13). The original questionnaire was modified by adding questions on early childhood development and diabetes management.

Subjects were administered the Child Attachment Interview (CAI) (14), a narrative-based assessment designed to elicit children's internal working models of attachment. Unlike semi-projective instruments, the CAI is a direct interview, calling on children to describe and reflect on their current attachment relationships and experiences. The interview is intended for use with 8–15-year-olds. It is assessed by analyzing the transcripts and video analysis of behavior for the presence of attachment disorganization. The subjects are then classified with respect to the relationship with each attachment figure either into two main groups (two-way classification: secure or insecure), three main groups (three-way classification: secure, preoccupied, or dismissing), or four main groups (four-way classification: secure, preoccupied, dismissing, or disorganized) (14, 15). CAI protocols were evaluated by three independent coders, all accredited by one of the CAI authors. Inter-rater reliability was conducted on 60 interviews for pairs of raters (20 interviews for each pair).

Children completed the Lifetime Incidence of Traumatic Events—Student Form (LITE-S) (16) to gauge exposure to adverse life events. The 16 items cover a broad range of potential trauma and loss events and ask for an estimate of emotional impact at both the time of occurrence and at the present. The questionnaire is available in student and parent forms (LITE-S, LITE-P), and its test–retest reliability for the total scale was found to be 0.76, and kappa per item ranged between 0.33 and 0.86 (16). LITE-S has been officially translated and validated in the population of Slovene primary school students (unpublished data). We used the LITE-S All Events scale, reporting the cumulative number of stressful events.

The subjects' carers were administered the Social Readjustment Rating Scale (SRRS) questionnaire regarding stressful life events in the family from pregnancy to the present (17). The questionnaire was adjusted to assess 4 different life periods (from pregnancy to first year of life, the year before the diagnosis of T1D/before entering school for the controls, the past year, anytime in the child's life). Gerst et al. (17) tested the reliability of the SRRS by re-administration of the test after 3, 6, 12, and 24 months, and found high temporal consistency for healthy adults (*r* = 0.96–0.89) and psychiatric patients (*r* = 0.91–0.70). The questionnaire was previously translated and has been widely used in the Slovene population.

Carers' attachment patterns in close relationships (to each of their parents, to a partner, to a best friend, and general attachment) were assessed by the Relationship Structures Questionnaire (ECR-RS) (18). The scale assesses attachment to each of the four attachment figures by the same nine questions, and the general attachment is calculated as a mean of the results for all the figures. If the score for one of the attachment figures was missing, the mean was calculated from the remaining scores. Within each relational domain, the questionnaire assesses two dimensions: attachment-related anxiety (how worried the person is that the attachment figure may reject him or her) and attachment-related avoidance (what kind of strategies the person uses to regulate their attachment behavior in the relational context, from being comfortable using others as a secure base and safe haven, to being uncomfortable with closeness and dependency) (7). The securely attached person scores low on both of these dimensions. The reliability for the dimensions is from high to excellent (Cronbach's alpha above 0.7 for different attachment figures and domains) (18).

### Data Analysis

The descriptive comparisons between two groups (cases vs. controls) were made using independent samples *T*-test, Mann–Whitney *U*-test, Pearson chi-square test, and Fischer's exact test predictor in the statistical package IBM SPSS for either continuous or categorical variables. The multivariate binomial logistic regression model in statistical program R was employed to predict T1D outcome depending on different predictors. The predictive variables used in the models were the following: (1) a two-way attachment classification (CAI), (2) attachment-related anxiety in carers (ECR-RS-anx-carers), (3) attachment related avoidance in carers (ECR-RS-avoid-carers), (4) stressful life events in the family during pregnancy with the child and the first year (SRRS_1y), (5) stressful life events in the family in their lifetime (Lifetime SRRS), (6) stressful life-events in the child (LITE-S), and (7) mothers' education level. Four models were tested in which different variables were used to predict T1D (primary outcome). Two-way attachment classification and stressful life events reported by children and carers were used in the first model; two-way attachment classification and stressful life events reported by carers in the second model; attachment-related anxiety in carers, attachment related avoidance in carers, and stressful life events reported by children and carers in the third model; and the mother's education level was added as the predictor in the fourth model. We decided to add the mothers' educational level to the fourth statistical model since there was a statistically significant difference between the groups (cases and controls). Education was used as an ordinal variable (level of education: 1 = unfinished primary school, 2 = primary school, 3 = unfinished high school, 4 = high school, 5 = unfinished college, 6 = college, 7 = unfinished university, and 8 = university) and then three levels were analyzed according to median and quartiles. The differences were considered statistically significant for a *p*-value at or below 0.05.

All of the independent numerical scale variables in the binary multivariate logistic regression model were centered and both independent categorical variables were dummy coded. The logistic regression model was created with the backward stepwise procedure (19), where all of the second order interaction terms of the independent variables were included in the beginning. Thus, the final refined logistic regression model included only the main and the interaction terms of which removal would worsen the model.

## Results

### Participants

**Figure 1:**
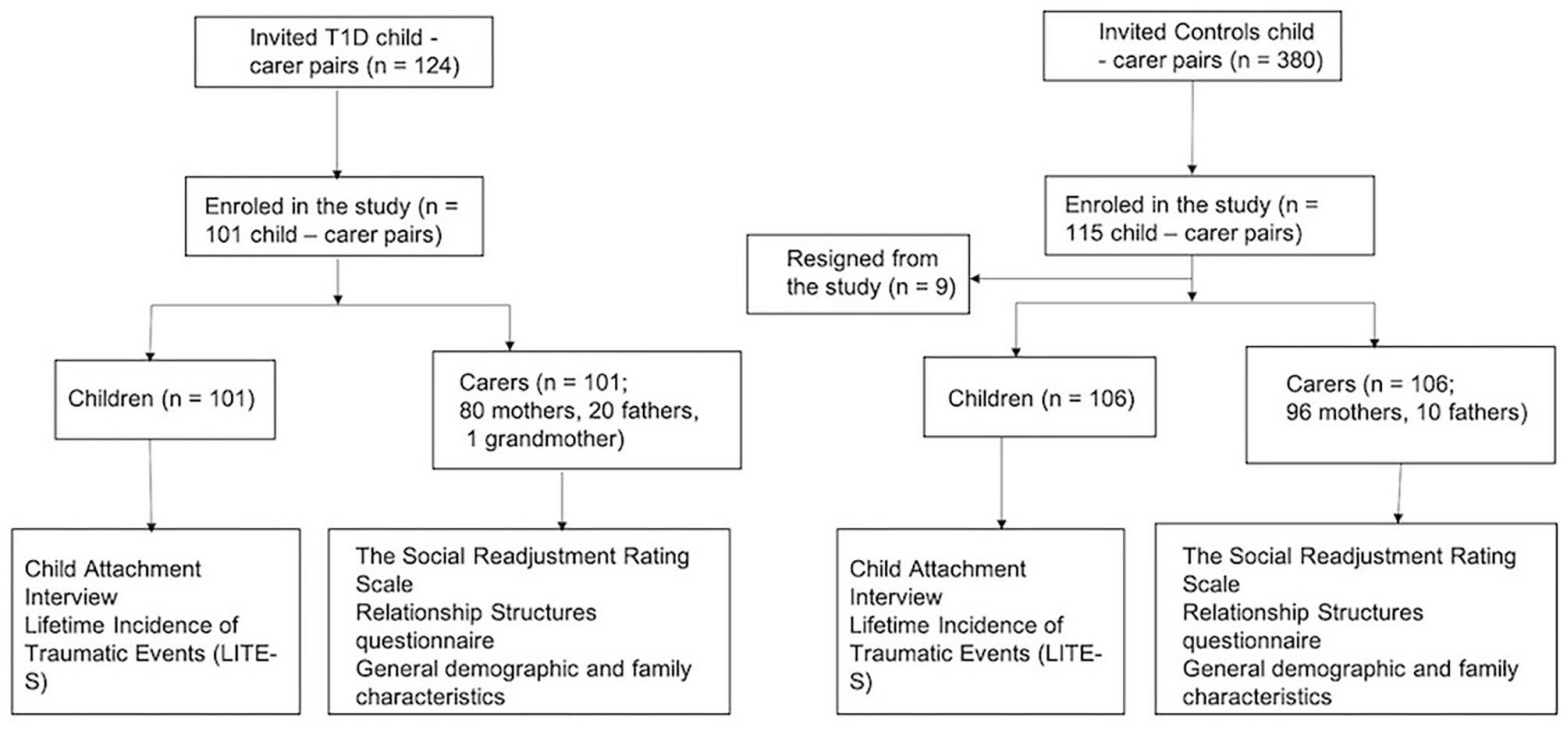
The consort flow diagram.

One hundred and twenty-four families of children in the Slovenian national childhood-onset T1D register were invited to participate, of which 101 agreed to participate. Of the 380 pupils invited to be in the control group, 115 agreed to participate, with nine families dropping out shortly after the start of the study; 106 healthy pupils/families completed the study. The participation rate in the research group was 81.5% and in the control group 27.9% (Figure 1 presents CONSORT Flow Diagram).

The study included 207 children, aged 11.7 ± 2.1 years; 109 were females. Of these 101 had T1D (aged 11.8 ± 2.1; 50 females), and 106 were in the control group (aged 11.6 ± 2.1; 59 females). The average duration of T1D was 5.2 ± 3.4 years. The majority of children with T1D (81.3%) were using an insulin pump. In the research group, the questionnaires were filled out by mothers in 79.3%, fathers in 19.8%, and other carers (grandmother) in 1%. In the controls, there were 90% mothers and 10% fathers. Baseline characteristics are presented in Table 1.

**Table 1:** Demographic data and results of the measured attachment to the mother, child stressful life events, family stressful events, and carers' attachment for children with T1D and healthy controls.

	**Cases T1D**	**Controls**	***p***
	**(*N* = 101)**	**(*N* = 106)**	
Age in years	11.8 ± 2.1	11.6 ± 2.1	0.656
Female gender	50 (49.5)	59 (55.7)	0.375
Duration of T1D in years	5.2 ± 3.4	/	/
Age of mother in years	41.1 ± 5.1	42.3 ± 4.4	0.112
Age of father in years	43.9 ± 6.3	45.1 ± 5.7	0.053
Divorced family/living with one parent/living outside the family	*N* = 96	*N* = 102	
	19 (19.8)	15 (14.7)	0.251
Mother education level	*N* = 93	*N* = 101	**0.0001**
Finished secondary school	48 (51.6)	18 (17.8)	
Finished University	22 (23.7)	70 (69.3)	
Father education level	*N* = 93	*N* = 98	**0.0001**
Finished secondary school	57 (61.3)	32 (32.7)	
Finished University	15 (16.1)	45 (45.9)	
Mother employed	*N* = 94	*N* = 99	
	80 (85.1)	93 (93.9)	0.068
Father employed	*N* = 93	*N* = 98	
	81 (87.1)	92 (93.9)	0.600
CAI	*N* = 101	*N* = 106	
CAI Secure (4-way)	65 (64.4)	56 (52.8)	0.093
CAI Insecure (4-way)	36 (35.6)	50 (47.2)	0.093
Dismissing	25 (24.7)	29 (27.4)	0.670
Preoccupied^†^	0^†^	0^†^	
Disorganized	11 (10.9)	21 (19.8)	0.076
Lifetime SRRS	*N* = 95	*N* = 101	
	215.3 ± 200.7	220.7 ± 237	0.644
Median (IQR)	170.5 (221)	154.0 (260)	
SRRS pregnancy-first year	*N* = 95	*N* = 101	
	17.2 ± 40.4	28.1 ± 63.8	0.077
Median (IQR)	0.00 (0)	0.00 (25)	
LITE—S	*N* = 95	*N* = 101	
	2.7 ± 1.8	2.9 ± 1.8	0.375
ECR-RS	*N* = 93	*N* = 101	
ECR-RS-avoid-carers	2.6 ± 0.9	2.5 ± 0.9	0.841
ECR-RS-anx-carers	1.8 ± 0.9	1.5 ± 0.7	0.076

*Data are n (%) or Mean ± SD unless stated otherwise. The descriptive comparisons between two groups were made using independent samples T-test, Mann–Whitney U-test, Pearson chi-square test, and Fischer's exact test, statistical significance p < 0.05 (bold). CAI, Child Attachment Interview; SRRS, Social Readjustment Rating Scale; LITE-S, Lifetime Incidence of Traumatic Events—Student Form All Events scale; ECR-RS, Relationship Structures questionnaire; ECR-RS-avoid-carers, carers' attachment avoidance; ECR-RS-anx-carers, carers' attachment anxiety*.

† *One (1%) of the cases and three (2.8%) of controls were classified as preoccupied in the 3-way classification but all of them were disorganized in the 4-way classification*.

There were no statistically significant differences between the cases and the controls regarding age, gender, parent's employment, the security of attachment (CAI), presence of disorganization (CAI), lifetime stressful events in the family from the pregnancy of the child to child's first year or anytime in life (SRRS), lifetime stressful events as reported by the child (LITE-S), and carers' attachment (ECR-RS general anxiety or general avoidance). The groups were statistically significantly different in terms of parents' education level (Table 1). Namely, the education level of the parents in the control group was significantly higher than in the cases (see Table 1). Spearman correlation coefficient indicated that there was a moderate positive statistically significant correlation (rho = 0.629; *p* < 0.001) between the education levels of both parents. The inclusion of both parents' education in the model would therefore result in multicollinearity between the independent variables. Consequently, only the mother's education was used as a predictor in the subsequent statistical analyses.

Inter-rater reliability conducted on 60 interviews for pairs of raters was high for all main categories for mother (M) and father (F): for two-way M (*Kappa* = 0.89, *p* < 0.05), for three-way M (*Kappa* = 0.887, *p* < 0.05), for four-way M (*Kappa* = 0.708, *p* < 0.05), and all classifications F (*Kappa* > 0.7, *p* < 0.05).

Concordance between attachment to the mother and father was high, with 95.0% of children having the same two-way classification for both parents, 92.6% in the three-way, and 91.6% showing agreement in the four-way classification. Due to the high concordance and the fact that most of the material was provided by the mothers, we used only attachment to the mother in the logistic regression models.

Of the T1D cohort, 64.4% were classified as secure with their mother, and 35.6% as insecure in the three-way classification (34.6% dismissing, 1% preoccupied). In the controls, 54.7% were classified as secure with their mother and 45.3% as insecure in the three-way classification (42.5% dismissing, 2.8% preoccupied). The differences were not statistically significant.

### Primary Outcome

A binomial logistic regression was performed to ascertain the effects of two-way attachment classification (secure-insecure) in children or two-dimensional carers' attachment representation (anxiety-avoidance) and co-variates (stressful life events measured by SRRS from pregnancy to the end of the first year of the child's life or anytime in the family's life and stressful life events measured by LITE-S) on the presence of T1D (primary outcome).

In the first two models, the child's attachment to the mother (CAI two-way classification) with stressful life events from pregnancy to the child's first year or anytime in the family's life (lifetime SRRS) was not statistically significantly associated to child's T1D. The independent variables showed no statistically significant effects on the dependent variable (*p* > 0.05).

In the third model, carers' attachment anxiety was statistically significantly related to T1D (*p* < 0.05; *R*^2^ = 0.0613), namely the higher the attachment anxiety, the higher the association with T1D in the child (Table 2). The model explained only 6.1% of the variance.

**Table 2:** Results of the binary multinomial logistic regression model: carers' attachment anxiety/avoidance and stressful life events.

**Names**	**Estimate**	**exp(B)**	***z***	***p***
**Parameter estimates**				
(Intercept)	0.44025	1.553	11.359	<0.001
ECR-RS-anx-carers	0.13705	1.147	2.556	**0.012**
LITE-S	0.03400	1.035	1.454	0.148
SRRS_1y	−0.00104	0.999	−1.508	0.134
ECR-RS-avoid-carers	−0.03141	0.969	−0.708	0.480

*ECR-RS-anx-carers, carers' ECR-RS general anxiety; ECR-RS-avoid-carers, carers' ECR-RS general avoidance; LITE-S, Lifetime Incidence of Traumatic Events—Student Form; SRRS_1y, stressful life events from pregnancy to the end of first year. Lifetime SRRS, which was initially included in the model, was removed due to the lower statistical significance comparing to the other variables, a process of backward stepwise procedure that is thus not shown in the table*.

*Statistical significant values are bolded*.

In the fourth model where mothers' education level was added as one of the predictors, the following variables were statistically significantly associated with the child's T1D: child's stressful life events, carer's attachment anxiety, mother's education level, and the interaction between the latter two. The higher the score on the LITE-S, carer's attachment anxiety, and the lower the mother's education, the higher the association with the child's T1D. Table 3 shows the model in which mothers' level of education was added as one of the predictors [$X_{\left(10\right)}^{2}$ = 54.1, *p* < 0.001; *R*^2^ = 0.293]. The model explained 29.3% of the variance.

**Table 3:** Results of the binary multinomial logistic regression model: carer's attachment-related anxiety/avoidance and stressful life events—mothers' education level was used as one of the predictors.

**Names**	**Estimate**	**exp(B)**	***z***	***p***
**Parameter estimates**				
(Intercept)	0.4581	1.581	126.980	<0.001
SRRS_1y	−5.08e−5	1.000	−0.0638	0.949
LITE-S	0.0481	1.049	22.646	**0.025**
ECR-RS-anx-carers	0.1038	1.109	20.147	**0.046**
ECR-RS-avoid-carers	−0.0288	0.972	−0.7202	0.473
Education	−0.1191	0.888	−62.175	**<** **0.001**
SRRS_1y ^*^ LITE-S	−4.79e−4	1.000	−13.965	0.165
LITE-S ^*^ ECR-RS-anx-carers	−0.0536	0.948	−17.304	0.086
LITE_S ^*^ ECR-RS-avoid-carers	0.0524	1.054	19.333	0.055
ECR-RS-anx-carers^*^ Education	0.0582	1.060	23.938	**0.018**
ECR-RS-avoid-carers^*^ Education	−0.0350	0.966	−16.680	0.097

*SRRS_1y, stressful life events from pregnancy to the end of first year; LITE-S, Lifetime Incidence of Traumatic Events—Student Form; ECR-RS-anx-carers, carers' ECR-RS general anxiety; ECR-RS-avoid-carers, carers' ECR-RS general avoidance; Education, mothers' education level*.

* *, represents interaction between two independent variables*.

*Statistical significant values are bolded*.

**Figure 2:**
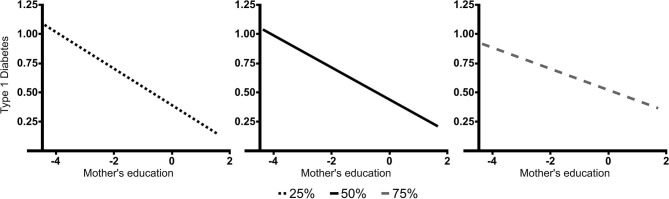
The relationship between mothers' education level and children's T1D for different levels of carers' attachment anxiety. The figure shows significant slopes for the three levels of carers' attachment anxiety. The lower the mother's education, the higher the association with T1D for all levels of carers' attachment anxiety. Type 1 diabetes—type 1 diabetes in children; Education—level of mothers' education (−4—finished high school or less, 0—finished university); 25, 50, and 75%—levels of carers' attachment anxiety on ECR-RS (divided in three groups according to quartiles).

**Figure 3:**
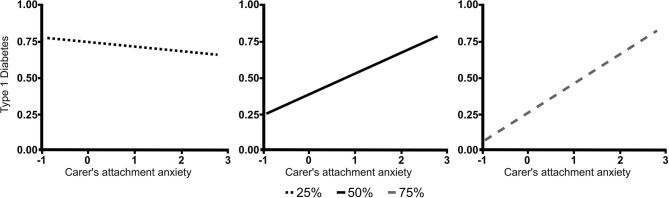
The relationship between carers' attachment anxiety and children's T1D for different levels of mothers' education. The figure shows significant slopes for the three levels of mothers' education. In the mothers with higher than secondary education, higher level of carers' attachment-related anxiety was positively associated to T1D in children. This was not the case for mothers with secondary or lower education. Type 1 diabetes—type 1 diabetes in children; Carers' attachment anxiety—carers' ECR-RS general anxiety; 25, 50, and 75%−7 levels of education presented as median and quartiles (25%—finished high school or less, 50%—unfinished or finished college, unfinished university, 75%—university).

The results based on the simple slope analysis showed that the lower the mother's education, the higher the association with T1D for all levels of attachment anxiety (Figure 2). However, a higher level of attachment-related anxiety in carers was also positively related to T1D in children. This was the case for mothers with higher than secondary education level (*p* = 0.007 for finished University and *p* = 0.01 for unfinished or finished college, unfinished university), and not for mothers with lower education levels (Figure 3). Model *R*^2^ = 0.293.

## Discussion

Our results showed that there was a statistically significant association between carers' (most often mothers') attachment anxiety and T1D. After adding mothers' education level to the model, the results showed a significant positive association between children's T1D and stressful events experienced by children, as well as carers' attachment anxiety. The positive association between carers' attachment anxiety and children's T1D was significant for mothers with higher education but not for mothers with secondary or lower education, with our model explaining 29% of the variance. We did not find a statistically significant association between children's attachment security and T1D.

To our knowledge, only one study to date has explored the relationship between carers' attachment and the occurrence of T1D in children. Sepa et al. investigated the relationship between maternal attachment (measured by the Adult Attachment Interview) and diabetes-related autoimmunity during infancy in 18 mothers of positive and 32 mothers of infants negative for diabetes autoantibodies. Whilst no statistically significant association was found, the results showed a higher proportion of insecure mothers in the autoantibody positive group (12). The development of T1D in children of caregivers with high attachment anxiety could be mediated by the caregivers' lower capacity for stress regulation, which in turn can lead to their reduced capacity for regulation of their child's stress response. This would be most acute at times wherein the attachment system is activated (perceived danger to the child) (20, 21). Carers with higher level of attachment related anxiety might show overconcern in relation to their children and thus upregulate instead of downregulating stress experienced by their children (21). Due to the observational design of the present study, it is also possible that attachment-related anxiety in carers could be a result of the stress related to the chronic illness of their children and the constant threat of possible serious medical complications related to it (22). The relationship of attachment-related anxiety and T1D could thus be seen as bidirectional (23).

Our results showed that the mothers' educational level negatively correlated with the probability of T1D: the higher the educational attainment, the lower the association with the child's T1D. The research of Virk et al. (24) on a large population-based sample showed an association between bereavement (loss of a mother, father, or sibling from age 5 years onwards) and increased rate of T1D when exposure onset began after 11 years of age. This association only persisted among children born to mothers with low educational attainment. They suggest that a lower maternal education level might be associated with increased vulnerability. These results, however, should be interpreted with caution, since the groups in our study differed in the parents' educational level in the first place and could be a result of selection bias.

Our results showed a statistically significant association between stressful life events in children (but not in the carers) and T1D in the model in which carers' attachment was the predictor of T1D. Therefore, our results partly support previous studies that showed a positive correlation between stressful life events in the life of parents or children and T1D-related autoimmunity or occurrence of T1D (8, 25). In a sample of 5,986 children and their families (the prospective population-based All Babies in Southeast Sweden project), Sepa and colleagues showed that maternal experience of serious life events (such as divorce or violence) was involved in the induction or progression of diabetes-related autoimmunity in children at age 2.5 years, independent of a family history of T1D (6). In the same project, childhood experience of a serious life event was associated with a higher risk of a future diagnosis of T1D (8). One possible explanation for our results might be the retrospective and not prospective nature of the stressful life-events evaluation, so the subjectivity and the change in certain experiences through time must be taken into account (26).

Our findings did not show a significant association between attachment insecurity in children and T1D. Thus, the hypothesis that insecurity of attachment in children represents a stress-related vulnerability and as such means, a greater risk for T1D was not confirmed in the current study. To date, no other studies have supported this hypothesis. Given that attachment representations are susceptible to change with time, being less stable in childhood than adulthood (7, 27), it is also possible that some children assessed as secure at the time of the study may have been insecurely attached before they developed diabetes. The availability, consistency, and responsiveness of medical professionals, such as the diabetes pediatrician (24-h phone availability) and the availability of care offered by other professionals from the diabetes team (nurses, educators, dietitians, psychologists, child psychiatrists, group and family therapists), could provide a safer environment for the families of children with T1D. The latter is also supported by previous reports of lower suicidality in adolescents with T1D (from the same hospital) compared to healthy adolescents (13).

The strengths of this study were the inclusion of almost the entire cohort of Slovene children with T1D within the specified age range, using an interview measure instead of a self-report for the assessment of child attachment, and large sample size as compared to similar studies. Using a case-control design enabled us to compare and also control for possible bias of the effect of different predictors on the primary outcome. The first limitation is the observational approach of the study. Even though the case-control design enabled us to control for the bias, from our sample we can only determine the associations, not the causality of the observed factors in the development of T1D. Even though we gathered very high-quality information on attachment in the children, the carers' attachment was only assessed by a self-reported questionnaire. Nevertheless, the questionnaire itself proved highly reliable (18) and our results add considerably to previous studies on the influence of parents' attachment (assessed by Adult Attachment Interview) to diabetes-related autoimmunity in children (12). The third limitation was the different recruitment and resulting differences in participation rates between the groups. This could have had an effect on the type of families who were part of the study and is also reflected in the differences in the parents' education levels. The groups did not differ in any other demographic or studied factors and the models were designed to overcome possible biases.

In conclusion, our findings suggest that some of the risk factors associated with T1D in children may be reduced by helping families under higher levels of stress, especially if there are indices of high attachment anxiety in the carers. Offering support for families early in the caregiving process, bearing in mind the role of the caregivers in children's stress regulation, would be a possible approach. Such interventions as well as longitudinal studies on the effects of parental attachment on the development of chronic disease in children should be evaluated in future studies.

## Data Availability Statement

The raw data supporting the conclusions of this article will be made available by the authors, without undue reservation.

## Ethics Statement

The studies involving human participants were reviewed and approved by the National Medical Ethics Committee of the Republic of Slovenia (No. 60/08/13). Written informed consent to participate in this study was provided by the participants' legal guardian/next of kin.

## Author Contributions

MD-R, SK, TB, NB, and YS-G designed the study. AT, KD, SK, NB, JL, KU, TB, YS-G, and MD-R were involved in the acquisition and interpretation of data. The first draft of the paper was written by AT, with the support of MD-R, who is the guarantor of this work and, as such, had full access to all the data in the study and takes responsibility for the integrity of the data and the accuracy of the data analysis. All authors contributed to conception of the work and approved the final version of the manuscript.

## Conflict of Interest

The authors declare that the research was conducted in the absence of any commercial or financial relationships that could be construed as a potential conflict of interest.
